# Prevalence and mobility of integrative and conjugative elements within a *Streptomyces* natural population

**DOI:** 10.3389/fmicb.2022.970179

**Published:** 2022-09-13

**Authors:** Caroline Choufa, Abdoul-Razak Tidjani, Anthony Gauthier, Manar Harb, Julie Lao, Nathalie Leblond-Bourget, Michiel Vos, Pierre Leblond, Cyril Bontemps

**Affiliations:** ^1^Université de Lorraine, INRAE, DynAMic, Nancy, France; ^2^Faculty of Medecine, CNRS, Grenoble INP, CHU Grenoble-Alpes, University Grenoble-Alpes, TIMC (UMR 5525), Grenoble, France; ^3^INRAE-ONIRIS, Nantes, France; ^4^INRAE, UR1404 MaIAGE, Jouy-en-Josas, France; ^5^European Centre for Environment and Human Health, Environment and Sustainability Institute, University of Exeter Medical School, Penryn, United Kingdom

**Keywords:** *Streptomyces*, integrative and conjugative element, gene transfer, population, genome, AICE, evolution

## Abstract

Horizontal Gene Transfer (HGT) is a powerful force generating genomic diversity in bacterial populations. HGT in *Streptomyces* is in large part driven by conjugation thanks to plasmids, Integrative and Conjugative elements (ICEs) and Actinomycete ICEs (AICEs). To investigate the impact of ICE and AICE conjugation on *Streptomyces* genome evolution, we used *in silico* and experimental approaches on a set of 11 very closely related strains isolated from a millimeter scale rhizosphere population. Through bioinformatic searches of canonical conjugation proteins, we showed that AICEs are the most frequent integrative conjugative elements, with the central chromosome region being a hotspot for integrative element insertion. Strains exhibited great variation in AICE composition consistent with frequent HGT and/or gene loss. We found that single insertion sites can be home to different elements in different strains (accretion) and conversely, elements belonging to the same family can be found at different insertion sites. A wide variety of cargo genes was present in the AICEs with the potential to mediate strain-specific adaptation (e.g., DNA metabolism and resistance genes to antibiotic and phages). However, a large proportion of AICE cargo genes showed hallmarks of pseudogenization, consistent with deleterious effects of cargo genes on fitness. Pock assays enabled the direct visualization of conjugal AICE transfer and demonstrated the transfer of AICEs between some, but not all, of the isolates. Multiple AICEs were shown to be able to transfer during a single mating event. Although we did not obtain experimental evidence for transfer of the sole chromosomal ICE in this population, genotoxic stress mediated its excision from the chromosome, suggesting its functionality. Our results indicate that AICE-mediated HGT in *Streptomyces* populations is highly dynamic, with likely impact on strain fitness and the ability to adapt to environmental change.

## Introduction

*Streptomyces* are mycelial and sporulating bacteria typified by a complex differentiation cycle and large linear chromosomes (Chater, 2016). They are ubiquitous in soils, are prolific producers of enzymes and secondary metabolites (Bérdy, 2005; Gavriilidou et al., 2022) and play central roles in biogeochemical cycles (Bontemps et al., 2013; van der Meij et al., 2017; van Bergeijk et al., 2020). *Streptomyces* populations can exhibit a remarkable degree of gene flux; a recent analysis of 11 *Streptomyces* clones isolated from a rhizosphere micro-habitat revealed that around a third of all genes were accessory, even though isolates had only very recently diverged (98.68%–99.99% core genome nucleotide similarity; Tidjani et al., 2019a,b). Horizontal Gene Transfer (HGT) mediating accessory gene gain and loss in *Streptomyces* is likely primarily mediated *via* conjugation, with only sporadic reports of natural transformation (Roelants et al., 1976) and transducing phages have only rarely been described ( Stuttard, 1979, 1983; Morino and Takahashi, 1997;Burke et al., 2001).

Conjugation is a conserved and major driver of the rapid evolution and adaptation of bacterial genomes (Grohmann et al., 2003) in a wide range of environments (Virolle et al., 2020; Groussin et al., 2021; Delgado-Blas et al., 2022). Conjugation is known to occur in soil (Koraki and Karagouni, 2000; Mafiz et al., 2021), and seems to be stimulated in niches where bacterial communities can reach high densities such as biofilms (Lopes et al., 2016; Luo et al., 2021) and plant associated niches including rhizosphere (Bañuelos-Vazquez et al., 2020a,b), phylloplane (Normander et al., 1998) and spermosphere (Sørensen and Jensen, 1998). Due to the high physical heterogeneity of soils and the high diversity of soil bacteria (Raynaud and Nunan, 2014), HGT in soil environments largely remains a black box. Moreover, conjugation is often narrowly seen through the prism of conjugative plasmids (Smillie et al., 2010; Kohler et al., 2019), but it has become clear that integrative conjugative elements are also very important (Andrews et al., 2018; Greenlon et al., 2019). Plasmid transfer has been assessed for *Streptomyces* in soil (Rafii and Crawford, 1988; Ravel et al., 2000), but HGT of integrative and conjugative elements has been rarely studied (Clerc and Simonet, 1998).

*Streptomyces* conjugative elements can be either extra-chromosomal (circular or linear plasmids) or chromosome-borne Integrative and Conjugative Elements (ICEs) and Actinomycete Integrative and Conjugative Elements (AICEs; Bordeleau et al., 2012; Chapleau et al., 2016). ICEs have been widely described in many Gram-negative and Gram-positive bacteria (Guglielmini et al., 2011; Bellanger et al., 2014). Their transfer starts by the excision from the chromosome by a site-specific recombinase (usually tyrosine or serine integrases) that recognizes specific direct repeat sequences (*att* sites) flanking the element. These direct repeats are the result of the action of the recombinase during the element insertion. Next, a relaxase nicks the circularized ICE at the origin of transfer (*oriT*). A coupling protein associated with the relaxase single stranded DNA complex allows its translocation to the recipient strain through a type IV secretory system (T4SS; Grohmann et al., 2018). After rolling circle replication and recircularization of DNA, the ICE will integrate in both the donor and recipient chromosomes at its *attB* target site. Closely related elements are Integrated and Mobilizable Elements (IMEs) that use similar excision and integration strategies but with non-related relaxases and coupling proteins. Their transfer is dependent upon a T4SS hijacked from another conjugative element (Libante et al., 2020).

The distribution of AICEs is limited to the phylum Actinobacteria. Integration and excision are identical to that of ICEs, but the AICE transfer mechanism is fundamentally different and depends on the TraB protein, a single membrane protein homologous to the chromosome segregation proteins during cell division and sporulation (FstK-SpoIIIE). The TraB transfer protein multimerizes into a homohexamer to form a membrane pore (Vogelmann et al., 2011). After excision and replication of the AICE, TraB specifically recognizes double-stranded DNA and translocates it into the recipient cell (Possoz et al., 2008; Vogelmann et al., 2011). While AICE replication continues in the donor and recipient cells, the element will integrate both chromosomes in a site-specific manner (Hagège et al., 1993). As for ICEs, duplication of the insertion site occurs during integration, generating direct repeats flanking the integrated element ranging from several nucleotides to hundreds of nucleotides in length. The AICE diffuses into the recipient mycelium through the action of Spd (spread) proteins (Thoma et al., 2019). This intra-mycelial spreading provokes a delay in development and sporulation of the receptor mycelium leading to the appearance of circular areas visible to the naked eye in agar cultures called ‘pocks’ (Kieser et al., 1982).

In this study, we take a joint bioinformatic and experimental approach to uncover the diversity of ICEs and AICEs and patterns of HGT in a sympatric *Streptomyces* population. We show that these elements are prevalent, diverse, and responsible for high levels of HGT and discuss how these elements contribute to the evolution and the adaptation of *Streptomyces* to their rhizosphere environment.

## Materials and methods

### ICE and AICE *in silico* detection

The identification in *Streptomyces* genomes of ICEs and IMEs was achieved using ICEScreen (unpublished), an automated and improved version of the two-way detection method developed by Ambroset et al. (2016) and Lao et al. (2020). Briefly, signature proteins corresponding to the recombination module (tyrosine or serine integrases) or the conjugation module (i.e., relaxase, coupling protein, VirB4 translocase of T4SS) are firstly identified with hidden Markov model profiles (HMM) from Lao et al. (2020) and secondly with BlastP searches (NCBI default parameters on nr database; Altschul et al., 1990) using protein tags from a curated database (Ambroset et al., 2016; Lao et al., 2020). In a similar strategy, AICEs were identified by the detection of signature proteins corresponding to the site-specific integrase (tyrosine or serine integrases), the replication function (Rep) and the transfer protein (TraB) with HMMsearch (Eddy, 2009) from the HMMER server (Finn et al., 2011) combined with blast search of known signature proteins (Ghinet et al., 2011) and HMM profiles (Mistry et al., 2021). HMM profiles (Supplementary Table S1) were either retrieved from Pfam 35.0 database (Mistry et al., 2021) or built from protein alignments for *Streptomyces* TraB (MUSCLE; Edgar, 2004) using HMMbuild from HMMERv3.3 with default parameters in Ugene platform (Okonechnikov et al., 2012). Identification of direct repeats flanking the element was manually performed by searching almost identical sequence patterns at the borders of each identified element. Cargo genes were assigned to functional categories (metabolism, DNA metabolism, resistance, regulation, phage, toxin/antitoxin, signaling, transposase and hypothetical proteins) using BlastP searches (E-value <10^−6^, coverage >80%).

### Sequence analysis and phylogenetic construction

All sequence analyses were performed with Ugene platform (Okonechnikov et al., 2012). MUSCLE alignments (Edgar, 2004) of protein sequences were performed with default settings and 3 iterations. Identity matrices were calculated based on previous alignments. A Neighbor-Joining tree was built with Mega X (Kumar et al., 2018). Synteny breaks between similar elements were identified by pairwise comparison with MAUVE (Darling et al., 2004). Comparison and visualization of the nucleotide identities along the integrated elements were performed using Artemis Comparison Tool (Carver et al., 2008) after BlastN alignment.

### Bacterial strains and culture conditions

The *Streptomyces* strains used in this study were isolated from mm-scale soil aggregates collected from a centimeter-scale rhizosphere population (Tidjani et al., 2019a,b; Supplementary Table S2). Strain storage, culture conditions and DNA extraction procedures were performed as described in Kieser et al. (2000). Briefly strains were grown on Mannitol Soya Flour medium (SFM) agar plates or grown in Hickey-Tresner (HT) medium liquid culture and mating experiments were performed on R2YE medium plates at 30°C. Kanamycin, apramycin and hygromycin antibiotics were used at 50 μg/ml (Kieser et al., 2000). Mitomycin treatments (125 ng/ml, corresponding to the quarter of the minimal inhibitory concentration) to induce ICE excision were performed in HT medium (OD 600 nm = 0.2) for 3 h (Volff et al., 1993; Bellanger et al., 2007).

### Strain labelling

To select recipient strains after mating, an antibiotic resistant gene (either *neo* for kanamycin or *aac (3) IV* for apramycin resistance) was inserted by homologous recombination at the chromosomal locus. The recombinant plasmid consisted of a pWED2 (Karray et al., 2007) derived suicide vector (this work) containing upstream and downstream homologous regions to the targeted locus flanking the hygromycin resistance gene (*hyg*). Labelled strains were obtained following a double crossover between plasmid-borne and chromosome homologous sequences and selection for resistance. Constructs were obtained by Overlap Extension PCR (Hilgarth and Lanigan, 2020) with primers listed in Supplementary Table S3. Constructs were introduced into *Streptomyces* sp. by conjugation with *Escherichia coli* ET12567/pUZ8002 (Kieser et al., 2000).

### Pock assays and validation of AICE transfer

Pock assays enabling directly visualization of conjugal AICE transfer were performed as described in (Huang et al., 2003). Briefly, 50–100 spores of the donor strain were spread on a lawn of the recipient strain (10^6^ spores) on R2YE medium plates. Pocks resulting from a growth delay of the recipient cells were observed by naked eye after 5–7 days of incubation at 30°C. At least three replicates were performed for each couple of donor and recipient strains. AICE flux between pairs of strains were represented using the CIRCOS tool (Krzywinski et al., 2009). To assess AICE transfer, clones were picked from pocks and streaked using sterile toothpicks on SFM media with kanamycin, allowing the selection of recipient strains. AICE transfers in streaks were confirmed by PCR using AICE-specific primers and primers targeting specific genomic sequences discriminating recipient and donor strains (Supplementary Table S3). To determine whether multiple AICE transfers resulted from the acquisition of multiple AICEs in a genome or from single transfers in different genomes, independent recipient clones were isolated from a streak in a second round of isolation and tested by PCR for AICE transfer.

## Results

### High diversity of conjugative elements in a rhizospheric population

**Figure 1:**
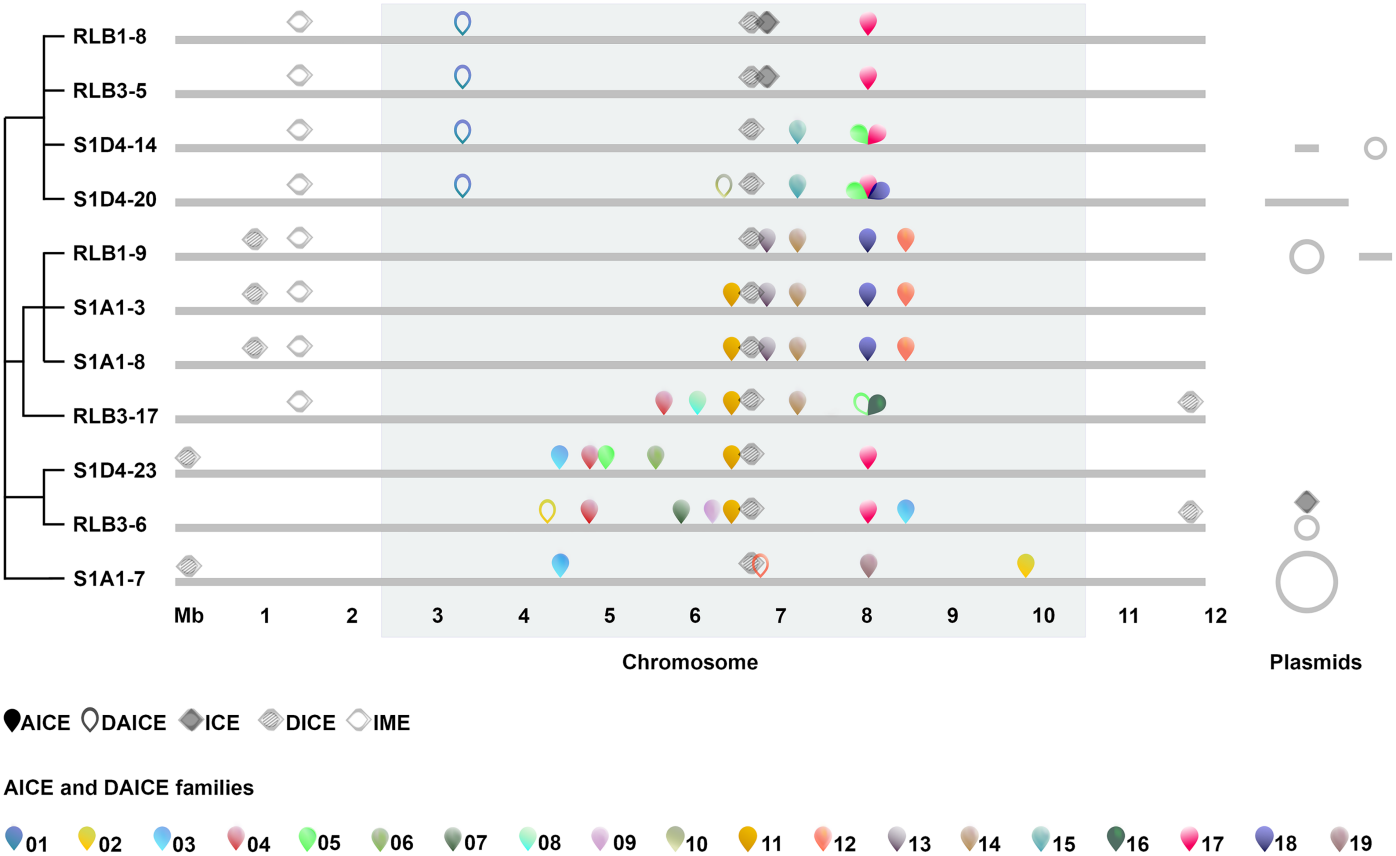
Prevalence and chromosomal distribution of conjugative elements in the *Streptomyces* population. Conjugative and integrative elements (AICEs, DAICEs, ICEs, DICEs, IMEs) are positioned along the chromosome (light grey line) of each isolate (relative to the RLB1-8 genome). The core-genome is highlighted in the grey band (2.4 and 1.4 Mb) are devoid of conserved genes on the left and right arms. The scale of 1–12 Mb corresponds to the genomic positions. ICEs, DICEs, and IMEs are represented by full, striped and empty diamonds respectively; AICEs and DAICEs are indicated by full and open pins, respectively, and each AICE family is represented by a color. Evolutionary relationships between strains are represented by a modified cladogram from Tidjani et al. (2020). Linear and circular plasmids when present are displayed on the right of the chromosome (note that representation is not proportional to their size).

Whole-genome sequences of 11, very closely related clones isolated from a mm-cm scale rhizosphere population were mined for the presence of conjugative elements. First, we screened for four markers of ICEs using the tool ICEscreen, namely the recombination module (tyrosine or serine integrases), the conjugation module (relaxase), the coupling protein (CP) and the VirB4 translocase of T4SS complex. The presence of all four functions was used to detect and classify ICEs, whereas elements that lacked T4SS were classified as IMEs. ICEs that have lost the integrase and/or the coupling protein and/or the relaxase were classified as defective ICEs (DICEs). Three putative ICEs were identified in the population (Figure 1; Supplementary Table S4). Two were found on the chromosomes of the closely related strains RLB1-8 and RLB3-5 (99.9% ANI) and not in their closest conspecific strains, which could be consistent with horizontal inheritance. Both encode 87 CDSs, share the same insertion site (*tRNA-Thr*) and produce the same terminal direct repeats upon integration into the chromosome (93 nucleotides). The third complete ICE was found on plasmid pRLB3-6.1 (present in the strain of the same name). It had an integrase distinct from that of the chromosomal element (no amino acid identity) and exhibited limited nucleotide similarity to the coupling proteins (48%), the relaxases (42%) and the virB4-like proteins (33%) of the chromosomal element. We identified three DICEs. One was close to the middle of the chromosome and present in all strains. One was shared by strains RLB1-9, S1A1-3, S1A1-8, and RLB3-17, this latter being located in the opposite arm compared to others. Similarly, the third DICE is shared by S1D4-23, S1A1-7, and RLB3-6, but in the opposite arm in RLB3-6. Finally, an IME was identified in all strains but RLB3-6, S1D4-23, and S1A1-7, with a single loss event in that clade being the most parsimonious scenario (Figure 1).

Second, we screened for AICEs using transfer protein (TraB) as a signature (Ghinet et al., 2011; Bordeleau et al., 2012) with HMM profiles and tBlastn searches. When elements thus identified possessed the integrase (*int*) and replicase (*rep*) signature genes, they were considered putatively functional and classed as an AICE. When the replicase and/or integrase was missing, the element was classed as a defective AICE (DAICE). A total of 51 *traB* genes flanked by direct repeats, were retrieved in the 11 chromosomes. TraB proteins showed a great disparity in sequence and in size (ranging from 139 to 738 amino acids). The four smallest versions of TraB proteins were associated with genes encoding an ATP/GTP-binding protein gene and a partitioning protein (ParM). Blast research systematically attributes the tripartite system ATP/GTP binding protein; short TraB; ParM protein to *Streptomyces* extrachromosomal elements. This could be an original chromosomal conjugation system derived from the integration of a conjugative plasmid. A phylogenetic analysis (Supplementary Figure S1) showed that the TraB proteins cluster with different *Streptomyces* TraB families (Ghinet et al., 2011), suggesting diverse evolutionary origins. We used a 95% amino acid identity threshold consistent with this clustering to group TraB proteins into 19 families, 7 of which were represented by only a single TraB protein (Supplementary Figure S1). All 51 *traB* genes identified on the chromosomes were flanked by direct repeats (2–94 nt in length) and delineated regions from 11.9 to 36.2 kb (Supplementary Table S5). Forty-three *traB* genes were associated both with a replicase and an integrase gene, suggesting a functional AICE. Eight remaining *traB* genes were not associated with functional replication or integration genes (six lacked replication signature genes, one lacked a canonical integrase gene and one harbored a transposon interrupting the integrase gene; Figure 1; Supplementary Table S5).

Four additional TraB ranging from 139 to 789 amino acids were found on extrachromosomal elements (data not shown). Because of their plasmid location, their divergence with chromosomal *traB* and the absence of direct repeats, they were discarded from further analyses although we cannot exclude the possibility that, these elements are functionally conjugative. Overall, each strain of the population harbored 5–10 elements (7.2 per genome on average), mostly represented by AICEs (Figure 1). In terms of size, IMEs/ICEs (86.0–99.7 kb) far exceed the size of the AICEs/DAICEs (11.9–36.3 kb), mostly depending on the presence of accessory genes (Supplementary Tables S4, S5).

### The central chromosome region is a hotspot for integrative element insertion

Functional chromosomal conjugative elements are exclusively distributed in the central region of the chromosome (Figure 1), i.e., in the region containing the 971 genes composing the core genome of the *Streptomyces* genus in between the left- (2.4 Mb) and right- (1.4 Mb) chromosomal arms which are devoid of conserved genes (Lorenzi et al., 2019). The insertion sites are located mostly in tRNA genes (*n* = 36) and also in protein-coding genes (*n* = 14) or intergenic space (*n* = 1; Supplementary Table S5). This preference of integration in such conserved targets could contribute to the high prevalence of elements in the core region. Only defective elements (one IME and two DICEs) were encountered in the arms. Their decay could result from intense recombination in these regions (Fischer et al., 1998; Choulet et al., 2006; Lorenzi et al., 2019). The subsequent formation of numerous insertions/deletions could make these elements non-functional until they eventually disappear from the genome.

### A single insertion site can host a diversity of integrative elements

Closely related strains show insertion sites that harbor the same element suggesting its vertical inheritance (Figure 1; Supplementary Table S5). For example, AICE family 13 is only shared by the strains of the same clade (RLB1-9, S1A1-3 and S1A1-8) and could have been acquired by their most recent common ancestor. On the other hand, some distributions (family 03, 11, 17, and 18) clearly suggest that the population is subjected to an intense flux of AICEs resulting from HGT and/or differential gene loss. For instance, AICE family 11 is present in several closely related strains (S1A1-3, S1A1-8, RLB3-17, S1D4-23, and RLB3-6) and is probably lost in strain RLB1-9. About one third of the elements (10 AICEs and 4 DAICEs) were present in only one strain, suggesting recent acquisition from unsampled donor strains. The fact that the insertion sites remain empty in some strains shows that they probably did not encounter the above elements and remain available for further gene acquisition.

**Figure 2:**
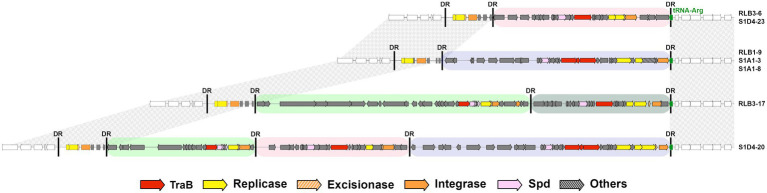
Accretion of AICEs and DAICEs at an insertion hot spot. An illustration of the diversity of conjugative elements within the same *tRNA-Arg* gene shared by different strains of the population (located *ca.* 8 Mb relative to RLB1-8 chromosome) as well as in some cases the multiple integration of several elements within this site (i.e., accretion). This is the case for strains RLB3-17 and S1D4-20 that harbor two and three functional elements, respectively. All strains share a conserved remnant element devoid of *traB* between their left-hand direct repeats (DR). The black vertical bars represent direct repeats, and the colored boxes represent the different AICE families (green: AICE05, grey: AICE16, pink: AICE17, purple: AICE18). Synteny on either side of the accretion site is indicated by textured grey areas. Genes are colored according to functions: inter or intra mycelial transfer, replication, excision/integration and others (legend below the figure).

In one case, several elements are integrated within the same insertion site (*tRNA-Arg*, Figure 2). One to three elements, AICEs or DAICEs belonging to three different families, are flanked by identical direct repeats. An additional trace of an ancient element, devoid of *traB*, is also present at this site. This phenomenon is called accretion (Bellanger et al., 2014) and corresponds to the successive integration of different elements having the same integration specificity. Notably, the different AICE families integrated at a single site (for instance *tRNA-Arg*) harbor different integrases (having 39%–88% of amino acid identity). Thus, there is no correlation between integrase sequence and integration site.

**Figure 3:**
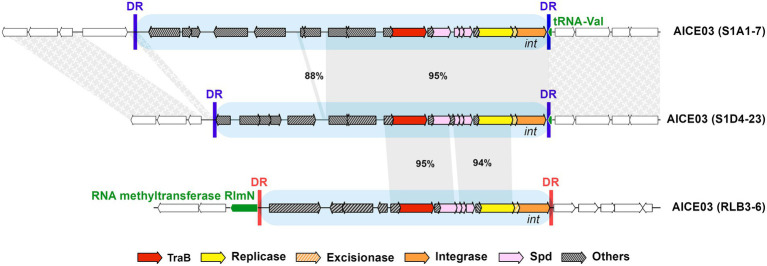
Intra-familial diversity in an AICE. An illustration of the gene content diversity of elements belonging to the same family (i.e., sharing the same highly identical TraB proteins), as well as the integration site specificity engendered by different integrases. These three AICEs belong to the same family (family 03 highlighted by blue boxes) but all share different cargo genes. While AICE03 in strains S1A1-7 and S1D4-23 share the same integrase and are inserted in the same *tRNA-Val* gene, AICE03 in RLB3-6 possesses a divergent integrase and excisionase and is found inserted in *rlmN*, a 23S methyltransferase encoding gene. The specific direct repeats generated by the integrase upon the element integration are represented by vertical bars and are different depending on the integrase and their target gene. Direct repeats (DR) of AICE03 in strains S1A1-7 and S1D4-23 are identical (blue), while those of strains RLB3-6 are different in size and sequence (red). The conserved regions between the AICEs are indicated by grey bands with percentage nucleotide identity indicated. Other examples of intra-familial AICE diversity are depicted in Supplementary Figure S2 and S3. Synteny on either side of the insertion site is indicated by textured grey areas.

In some cases, elements belonging to the same family can be found at different insertion sites, as illustrated by a family 3 element (Figure 3). The two AICE03 in strains S1A1-7 and S1D4-23 that share the same integrase are inserted in a *tRNA-Val* gene while AICE03 in RLB3-6 possesses a divergent integrase and excisionase and is found inserted in *rlmN*, a 23S methyltransferase encoding gene. The direct repeats flanking these elements are different in sizes and in sequences (46 nucleotides for S1A1.7 and S1D4-23 vs. 11 nucleotides for RLB3-6; Supplementary Table S5). Switching the integrase thus may provide a way to diversify the integration site of an element. In addition, the comparison of the association of the three signature genes (*traB*, *rep* and *int*) within a same family showed that exchanges of functional modules can occur between elements (Supplementary Table S6; Supplementary Figure S2), leading to changes in integration specificity as previously seen in mobile genetic elements (Garriss et al., 2009). While the integrase can be considered the driver of integration specificity, the same site can host elements with different integrases as demonstrated by the integration at the *tRNA-Thr* gene of family 13 AICE and the chromosomal ICE (Figure 1; Supplementary Table S5).

### AICEs are prone to decay

**Figure 4:**
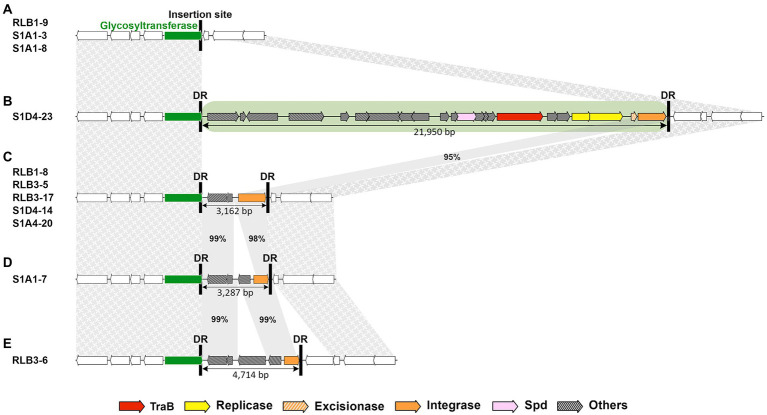
AICE decay. An example of AICE content at the same insertion site in different strains of the *Streptomyces* population. While some strains **(A)** harbored an empty site (in this instance, within a glycosyltransferase gene represented by the green arrow), S1D4-23 **(B)** possessed a fully functional AICE (family 06 highlighted in green). The strains depicted in **(C)**, **(D)** and **(E)** carry remains of cargo genes and truncated integrases between the direct repeats (black vertical bars; DR) inherited from an original AICE integrated at this site that have decayed to different degrees. Sequences upstream and downstream of the AICE insertion (including the empty site) is syntenic (textured grey areas), except for the gene encoding a transposase present in S1D4-23 and RLB3-6. The horizontal green arrow corresponds to the insertion site (phospholipid carrier-dependent glycosyltransferase). The size (in base pairs) of the AICE and decayed versions is specified below the elements. The grey bands show the conserved regions between the AICE and degenerate elements with the percentage of nucleotide identity.

Most AICEs exhibited high diversity in both cargo and mobility genes (Figure 3; Supplementary Figures S2, S3). Syntenic AICEs with identical gene content were only identified in very closely related strains (for instance for the family 01 of the cluster RLB1-8; RLB3-5; S1D4-14; S1D4-20 or family 14 of the RLB3-17; RLB1-9; S1A1-3; S1A1-8; Figure 1). Most of the diversity results from deletion and/or gene replacement occurring within the element. Recombination events resulting in losses of integrase or replicase genes are at the origin of DAICEs (Figure 4; Supplementary Figure S4). Some strains exhibit an empty integration site (RLB1-9, S1A1-3, and S1A1-8), when strain S1D4-23 had a genuine AICE (Figure 4). In other strains, only a partial integrase, cargo genes and two direct repeats [both related to those of the AICE06 (S1D4-23)] are suggestive of a former presence of AICEs at this site. It has to be noted that these latter could not be detected by our *in silico* approach and were here identified by the comparison of the genomes at the integration site of the AICE06 (S1D4-23). It is possible that the presence of flanking direct repeats could result in the future accretion of an AICE and a possible mobilization in *cis* of these remaining genes.

### AICE/ICE-borne cargo genes

**Figure 5:**
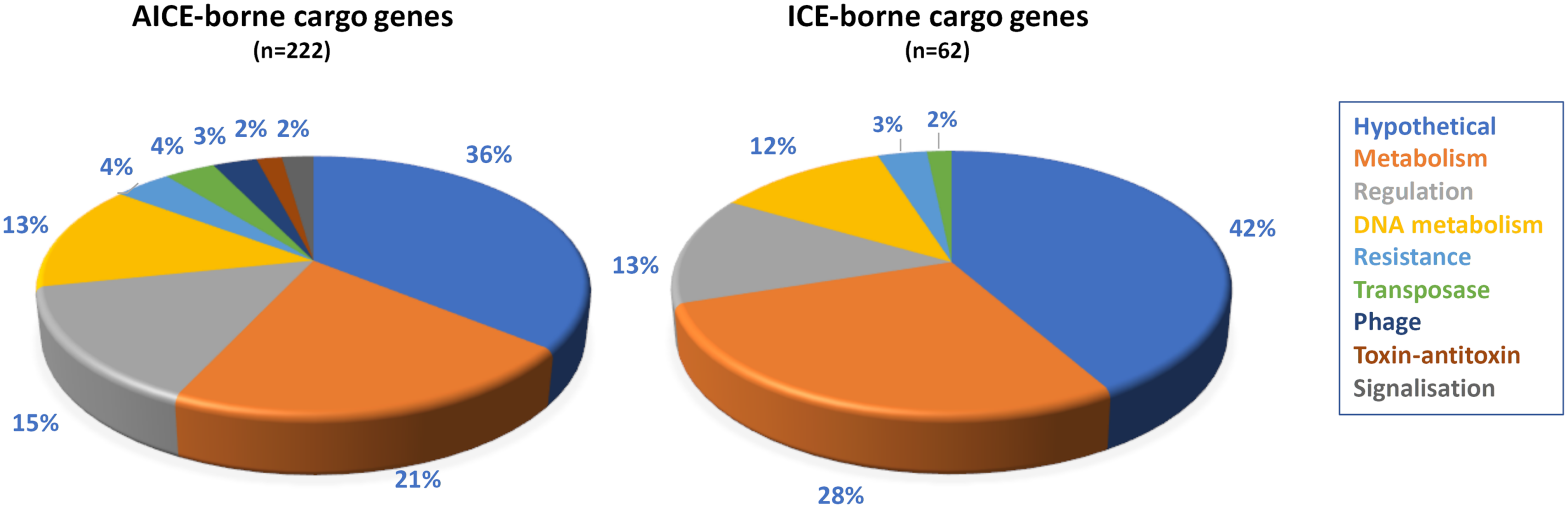
Functional distribution of the cargo genes carried by AICEs/ICEs. Cargo genes (protein size threshold >150 amino acids) were assigned to functional categories using BlastP searches (*e*-value cut-off 10^−6^, coverage threshold 80%). The functional categories are indicated by colors whose correspondence is indicated below the figure. BlastP results are presented in Supplementary Tables S7 (AICE), S8 (ICE).

Apart from genes mediating excision, integration, replication and intra-and inter-mycelial transfer, ICEs and AICEs also harbor cargo genes that can affect the fitness of the host cell. *In silico* analyses identified 541 unique genes in AICEs of which 445 are cargo genes. About 50% (223) of them were shorter than 150 amino acids, the threshold applied in our genome annotation process to get rid of probable pseudogenes (Tidjani et al., 2019a). The high percentage of putative pseudogenes in comparison to the rest of the genomes (*ca.* 7%), indicated that many AICE cargo genes are undergoing pseudogenization (Liu et al., 2004a). Furthermore, in order to identify as many functions as possible, a manual analysis of the shortest CDS was performed and enabled to identify the function of few conserved short proteins. The 222 proteins (*ca.* 50%) of size greater than or equal to 150 amino acids were defined as cargo gene products (Supplementary Table S7). Figure 5 shows the distribution of the 222 considered cargo genes in functional categories. For a subset of these genes, it is possible to assign (hypothetical) function. Genes that could be beneficial for the host cell included those implicated in antibiotic resistance (e.g., aminoglycoside phosphotransferase), cell signaling and metabolism (e.g., flavoprotein). Moreover, genes involved in illegitimate (encoding ligase) or homologous recombination (e.g., *radA*) which can provide additional or altered specific repair capacities or allow integration of foreign DNA (Supplementary Table S7) were identified. In addition, predicted protection systems against the entry of foreign DNA such as phages (e.g., Restriction and Modification or AIPR abortive phage infection systems) were detected. Toxin/antitoxin systems carried by some AICEs are likely addiction systems involved in the persistence of the mobile element, not expected to be beneficial to the cell (Song and Wood, 2020). The same analysis was performed on the 87 CDS of the chromosomal ICE which harbored 81 cargo genes of which 23% (*n* = 19) were shorter than 150 amino acids. Their assignment into functional categories was similar to that of AICE cargo genes (Figure 5; Supplementary Table S8).

### Multiple AICEs can transfer during a single conjugation event

**Figure 6:**
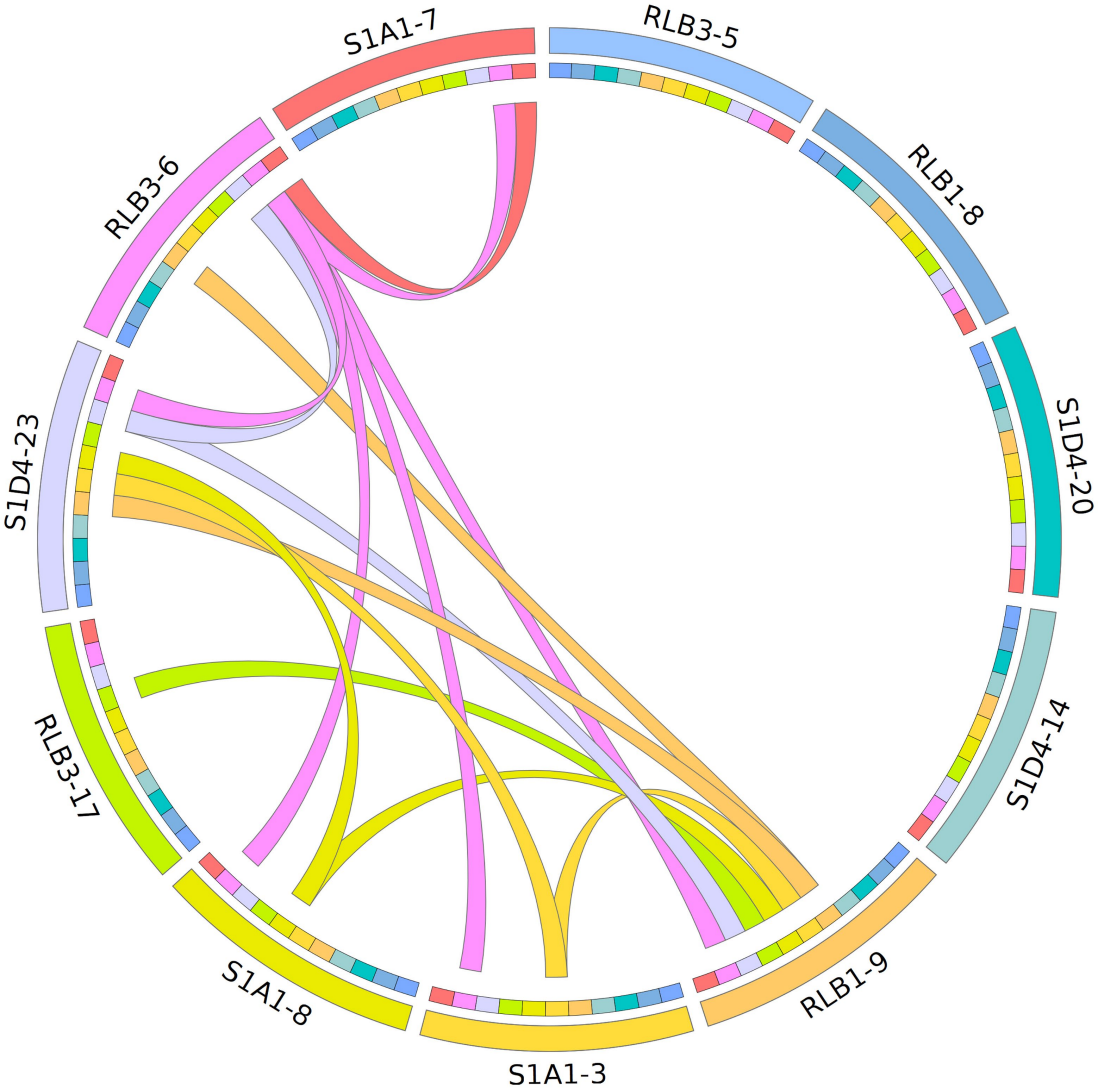
Mapping of AICE flux within the population based on a pock formation assay. Each of the 11 strain is represented by a colour (outer ring) and was tested as donor and recipient in bi-partite matings. The formation of pock is indicated by a link between two strains (interior ring). The colour of the link corresponds to that of the donor. The shades of colour symbolize clade affiliation as seen in Figure 1.

**Figure 7:**
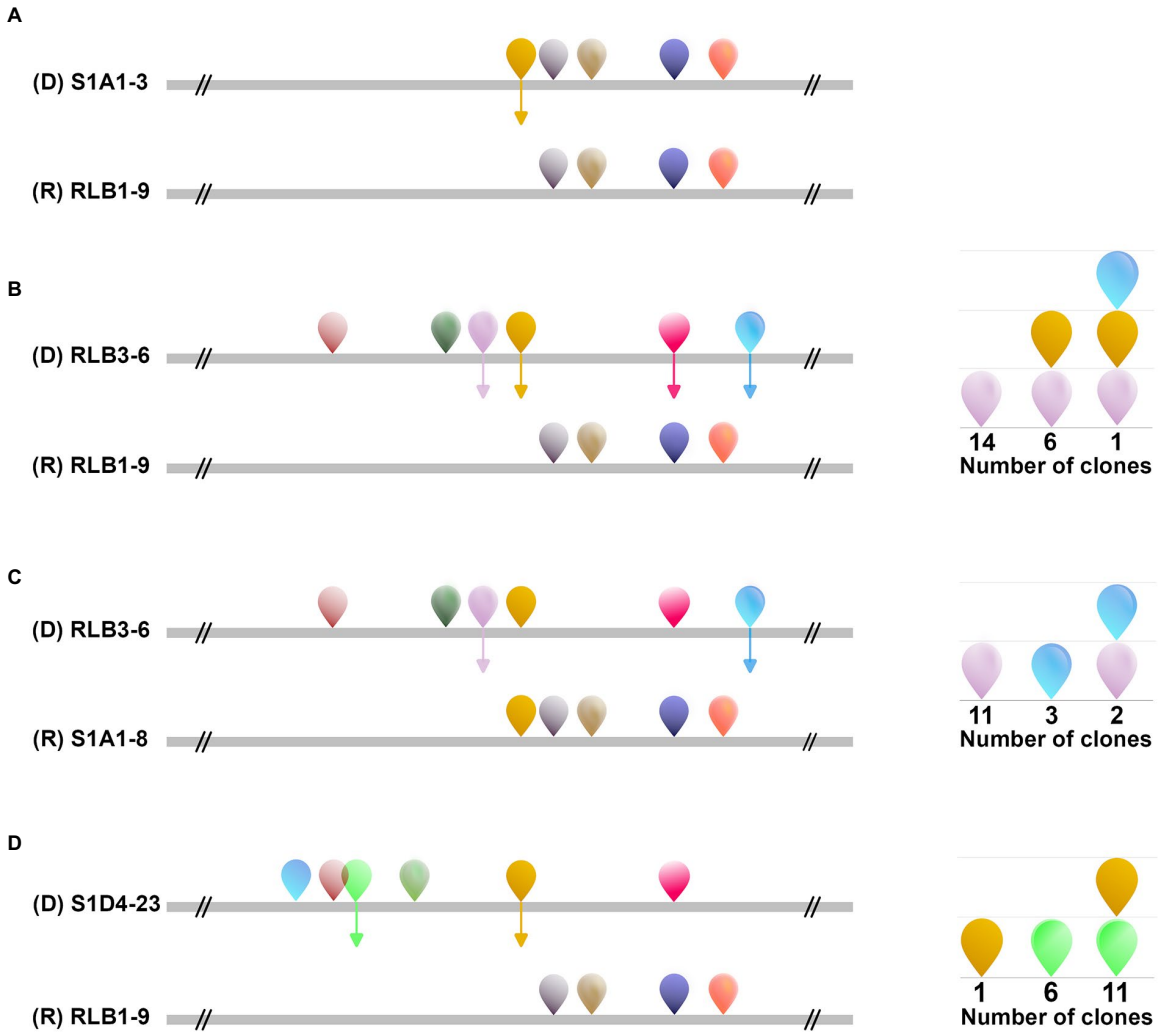
Multiple AICE transfer during a single mating event. The transfer spectrum of AICEs within pocks between four different strain couples [**(A)**: S1A1-3/RLB1-9, **(B)**: RLB3-6/RLB1-9, **(C)**: RLB3-6/S1A1-8 and **(D)**: S1D4-23/ RLB1-9] was assessed by PCR amplification of regions specific to each element. The strain represented at the top of each couple was the donor strain. The position and the family of the different elements present in both the donor and the recipient strains are schematized as in Figure 1. Depending on the couple, transfers from one to four different AICEs were identified, represented by the colored arrows. As a pock represents a population of different cells that could have experienced different AICE transfers, it was not possible at this stage to conclude whether multiple AICE transfers were the result of the concomitant transfer of several AICEs between the donor and a single recipient, or of multiple individual transfers between the donor and different recipient cells in the pock. After isolation of individual clones from a pock, both single and multiple AICE transfers were revealed in recipient cells. The schemes on the right-hand side represent which AICE was identified and revealed that a single strain can receive one to three different AICEs D: donor strain; R: recipient strain.

To assess the mobility of AICEs, we tested their conjugative ability by performing all pairwise mating combinations between the 11 strains (i.e., each strain was used as both a potential donor and a potential recipient). Conjugation events were assessed by direct visualization of pocks on mating plates. Pocks (2–5 mm) were observed in 15 out of the 110 tested pairwise combinations in seven strains that acted as donors, recipients or both (Figure 6). Horizontal gene transfer within pocks was screened by PCR for four selected mating couples (Figure 7; Supplementary Figure S5). These different couples represented situations with the potential to transfer one to six autonomous AICEs. The RLB3-6 donor strain harbored the plasmid pRLB3-6.1 on which no *spd* gene was identified, ruling out its participation to pock formation in our experiments. In a first step, we screened a mixture of cells picked from independent pocks. In one case, only one element was found to have transferred from the donor to the recipient in three out of three pocks (S1A1-3 × RLB1-9; Figure 7A) whereas in three other mating pairs several elements were found to have horizontally transferred within individual pocks (25 pocks for each couple; Figures 7B–D).

As a pock is composed of many different recipient cells, we could not conclude at this stage whether this phenomenon was the result of the concomitant transfer of several elements in a single cell, or of multiple transfers of single elements in independent cells. We therefore selected an independent pock showing evidence of transfer of multiple elements in three couples (RLB3-6 × RLB1-9; RLB3-6 × S1A1-8 and S1D4-23 × RLB1-9) and isolated independent recipient clones that undergone a sporulation cycle ensuring a unigenomic stage of the analyzed clones. Each of them was screened by PCR for each previously transferred element. In all three pairings, concomitant transfers from two to three AICEs were observed in independent recipient cells (Figure 7). Depending on the donor-recipient couple, multiple transfers occur in 13%, 33%, and 61% of the exconjugant clones.

### ICE01 can excise from its host genome

As ICE transfer is not accompanied by pock formation, the potential transfer of ICE01 was tested by genetically marking this element in strain RLB1-8 with apramycin resistance (see MM section). Mating of RLB1-8 with nine other strains marked with kanamycin resistance were performed, but no evidence of ICE transfer could be proved under our experimental conditions. As the excision of many chromosomally integrated elements such as phages or ICEs is repressed under lab conditions, their excision is often stimulated by genotoxic agents that induce the SOS response (Beaber et al., 2004). After exposure of the donor strain to the genotoxic antibiotic mitomycin C (125 ng/ml), the circularized ICE form could be detected by PCR analysis with primers targeting the *attP* sequence (data not shown) and the circularization was confirmed by sequencing the predicted *attP* sequence of 93 pb (Supplementary Table S4). Interestingly, a gene encoding a Helix-Turn-Helix DNA binding domain and a nudix hydrolase domain which could be related to the cI repressor family was identified on ICE01 (data not shown). Altogether these results suggest that this ICE element is functional and mobile under other conditions, as demonstrated for the TR ICEs in *S. turgidiscabies* (TR for toxicogenic region; Chapleau et al., 2016).

## Discussion

Our results reveal a high prevalence of conjugative and integrative elements within members of a microscale *Streptomyces* population, with no <80 elements (43 AICEs, 8 DAICEs, 3 ICEs, 18 DICEs, 8 IMEs) identified in the genomes of 11 very closely related strains. Genomes contained a multiplicity of conjugative elements (Bordeleau et al., 2012), with four AICEs on average per genome. We could confirm the preponderance of AICEs (*n* = 43) vs. ICEs (*n* = 3) in Actinobacteria as previously reported by Ghinet et al. (2011) and Bordeleau et al. (2012). Phylogenetic analysis of the AICE TraB protein sequences showed that the population host an AICE diversity almost as high as known for the entire *Streptomyces* genus (Supplementary Figure S1) and, except for two pairs of near-clonal strains in our population, no two strains harbored the same AICE content (Figure 1). Although these findings point at extensive gene flow in this population, the relatively limited number of genomes sequenced here did not allow to identify recent transfers between pairs of strains based on sequence analysis.

To directly test for conjugative horizontal gene transfer, we therefore used experimental bi-partite matings between these same strains. Only 15 pairs out of 110 formed pocks. Although the screening on the basis of mating pairs able of pock formation may underestimate the extent of gene flow within the population, our experimental results support our *in silico* observations on the importance of conjugation in population diversification. We also tested the possibility of observing pocks in crossings with more distantly related strains from the same micro-habitat (data not shown). Interestingly, evidence of conjugative transfer was obtained between strains from different species (ANI values <93.75%) as well as between strains belonging to the different *Streptomyces* main clades (Nicault et al., 2020). Thus, AICE transfer is not limited to phylogenetically related strains but can extend to other species inhabiting the same niche. Thus, if the insertion target sequence is present (and the site-specific recombinase active), the nucleotide divergence accompanying the phylogenetic distance might not be a barrier to the transfer of the element.

By analyzing the AICE content of exconjugants isolated from several pocks, we show that multiple AICE transfers can occur in a single mating. These results suggest either that multiple AICEs co-transferred in a single conjugational event or that sequential and independent transfers occurred and eventually ended up in the same recipient cell. In case of co-transfer, this raises several interesting questions, for instance, does each element use its own TraB-pore or can they hijack the TraB system of another element? This would imply a loss or absence of specificity of recognition of TraB for the elements. While TraB specificity was demonstrated in the case of conjugative plasmids pJV1, pSVH1, and pIJ101 (Franco, 2003; Vogelmann et al., 2011), no *clt* motif was described for the archetypal AICE pSAM2 although its transfer and regulation was well-documented (Possoz et al., 2008). Thus, the fact that several functional elements could transfer during a single conjugational event could either imply potential competition or cooperation between the elements themselves.

The experimental observation of horizontal transfer of single and multiple AICEs supports the notion of a high gene flow of conjugative events in *Streptomyces* populations. However, HGT events were not observed in seven out of the 11 strains, indicating that barriers to HGT must also exist. Whether these barriers are genetic [e.g., *via* restriction modification systems (Thomas and Nielsen, 2005)], exclusion systems (nudix; Possoz et al., 2003) or DNA modification such as phosphorothioation (Zhou et al., 2005) or ecological (e.g., *via* suboptimal abiotic or biotic conditions (Hanson et al., 2012) remains to be elucidated.

Although a large part of AICEs were predicted to be functional *in silico* (i.e., harboring the key functional signatures of replication, integration and transfer), some were identified as defective. These so-called DAICEs (defective AICEs) had the signature *traB* gene but lacked one or two of the three functional signatures but could remain mobilizable thanks to the presence of a helper element. This is likely when AICEs and DAICEs are integrated in tandem in the same site. In such accretion cases, the integrase of the functional element (AICE) can recognize its own flanking direct repeats or recognize those flanking the tandem AICE-DAICE and mobilize that composite element. Such *cis*-mobilization is well-documented in Gram-negative (Jacob and Wollman, 1958) and Gram-positive (Libante et al., 2019) but with few examples for Actinobacterial ICEs (Zhang and Loria, 2017). Another possibility that cannot be ruled out is that these apparently decayed elements are still mobilizable thanks to yet unidentified transfer functions. Whether the decaying processes (i.e., deletions, insertions) tend to eliminate integrated elements from the chromosome, our results indicate that exchange of functional modules between unrelated elements could lead to the creation of new combinatory elements (Burrus et al., 2002; Guérillot et al., 2013; Johnson and Grossman, 2015). We here uncovered examples of integrase recombination events between different elements enabling changes in integration specificity (although the replication, accessory and transfer modules can also recombine Supplementary Figure S2).

The vertical and horizontal transfer of mobile genetic elements represents a fitness cost to the host cell (Koonin et al., 2020) but this burden to the host can be counterbalanced by a putative advantage conferred by cargo genes, e.g., through antibiotic resistance (Khedkar et al., 2022) or symbiosis related genes (Sullivan and Ronson, 1998). We identified a significant number of cargo genes carried by AICEs. Although *circa* a third consisted of hypothetical proteins the function of a variety of cargo genes could be identified. These included candidates for ecologically relevant functions such as antibiotic resistance but also cargo genes whose potential benefits are less obvious, such as transcriptional regulators or restriction-modification (RM) systems. Future functional analyses and competition assays are needed to elucidate potential context-dependent fitness benefits of cargo genes. Interestingly, and consistently with ICEs (Liu et al., 2004b), there is a high level of pseudogenization in AICE cargo genes compared to genes in the core genome (7% vs. 42%). This is consistent with a scenario where the expression of cargo genes is not necessarily beneficial but often deleterious to the cell and only non-functional copies are maintained until they eventually disappear from the genome (Vos et al., 2015).

Another advantage to the presence of MGEs in genomes is their contribution to genome plasticity both by stimulating rearrangements and by promoting the acquisition of genetic information by horizontal transfer. For example, conjugative integrated elements dependent on a T4SS system are known to mobilize chromosomal DNA in *cis*, i.e., by physical association with the element. It is the regions adjacent to the insertion site of the element that can be mobilized into the receptor. This mechanism has been popularized with F-factor and directed transfer in *Escherichia coli* and discovered in many bacterial species (Hochhut et al., 2000; Daccord et al., 2010). In actinobacteria, the presence of ‘fertility factors’ (e.g., conjugative plasmids or AICE) is also associated with the formation of recombinants and therefore the transfer of chromosomal markers (Reeve, 1986). However, the amount of genetic information mobilized during a conjugative mating remains to be determined in *Streptomyces*.

## Data availability statement

The original contributions presented in the study are included in the article/Supplementary material, further inquiries can be directed to the corresponding author.

## Author contributions

CC, A-RT, MV, PL, and CB conceived and designed the project. CC, AG, and MH performed the experiments. CC, A-RT, JL, NL-B, and PL conducted the bioinformatic analysis. CC, MV, PL, and CB wrote the manuscript. All authors contributed to the article and approved the submitted version.

## Funding

CC thesis is granted by the Labex ARBRE and the Région Grand-Est (18_GE4_090). This work was supported by a grant overseen by the French National Research Agency (ANR) as part of the Investissements d’Avenir program (ANR-11-LABX-0002-01, Lab of Excellence ARBRE) and by the Région Grand-Est.

## Conflict of interest

The authors declare that the research was conducted in the absence of any commercial or financial relationships that could be construed as a potential conflict of interest.

## Publisher’s note

All claims expressed in this article are solely those of the authors and do not necessarily represent those of their affiliated organizations, or those of the publisher, the editors and the reviewers. Any product that may be evaluated in this article, or claim that may be made by its manufacturer, is not guaranteed or endorsed by the publisher.

## Supplementary material

Supplementary Figure S1 Phylogenetic tree of TraB proteins. A tree was built with 47 out of 51 TraB proteins identified in AICEs and DAICEs of the *Streptomyces* population. They were compared with 62 TraB sequences retrieved from Ghinet et al. (2011) that represent the TraB diversity found in sequenced *Streptomyces* species. TraB sequences of the population in the tree ranged from 433 to 738 amino acids. The four excluded sequences in the tree corresponded to TraB protein of family 18, which was too divergent and four very short sequences (139 amino acids) that could not be readily aligned. Plasmid-borne TraB (*n* = 4) were also excluded from the analysis. The unrooted phylogenetic tree was built with full length proteins using a Neighbor Joining method (JTT model) and percentage bootstrap support (100 replicates) is indicated if >70%. All positions with <50% site coverage were eliminated leaving a total of 567 positions in the final dataset. TraB sequences from our study are indicated with colored fonts. Their affiliation to different families, based on their phylogeny and sequence identity is indicated on the tree. The reference sequences are labeled with their accession numbers and according to the nomenclature of (Ghinet et al., 2011).

Supplementary Figure S2 Intra-familial diversity of mobility genes in an AICE family. The first four examples show AICEs with *traB* genes with a high identity (families 05, 04, 12 and 02 respectively), but with variability in their other mobility genes. The fifth example shows two AICEs with different TraB (families 16 and 19), but with closely related integrases and replicases. Legend is identical as in Figure 3. DR: Direct repeat.

Supplementary Figure S3 Intra-familial diversity of AICE cargo genes. Both examples show AICEs with similar mobility modules (*traB*, *rep* and *int*) with a high identity (families 11 and 18 respectively), but with variability in their cargo genes. Legend is identical as in Figure 3.

Supplementary Figure S4 Decaying process in AICEs. Illustration of the decaying process in the AICE04 family. Legend is identical as in Figure 4.

Supplementary Figure S5 Detection of AICE transfer in pocks. **(A)** Pictures on the left show mating plates for the strain couples S1A1-3 x RLB1-9; RLB3-6 x RLB1-9; S1D4-23 x RLB1-9 and S1A1-8 x RLB3-6. Examples of pocks are indicated with an arrow. Independent pocks were streaked on a selective recipient strain medium and AICE transfer was detected by PCR in each streak. For each couple, PCR results for 3 to 25 independent pocks are shown. The first or two top lines show PCR results performed with strain specific indels that discriminate donor and recipient strains. The two first columns are control PCRs performed with genomic DNA of the two parental strains. P1 to P25 indicate the PCR detection of indels and AICEs obtained from independent pocks. **(B)** Individual clones were isolated from a streak showing the transfer of several AICEs (framed on **A**) for couples RLB3-6 x RLB1-9; S1D4-23 x RLB1-9; S1A1-8 x RLB3-6. Multiple AICE acquisition in a single genome is indicated with an asterisk.

Supplementary Table S1 Pfam HMM profiles used for AICE and DAICE detection. The accession number of HMM profiles from the Pfam database and the description of the corresponding protein families were retrieved in Ghinet et al. (2011) except for TraB built in this study.

Supplementary Table S2 Strains and genome accession numbers.

Supplementary Table S3 Primers used in this work. Lower case letters represent 5′ end extensions of sequence not identical to the target (e.g., for addition of restriction sites). PCRs were performed on genomic DNAs (Kieser et al., 2000). PCR were performed with the DreamTaq (for visualization of AICE transfer, ICE transfer and ICE excision) or the Phusion (for strain labeling) kits with classical PCR conditions as described by the manufacturer (Thermo Fisher).

Supplementary Table S4 ICE and IME description. n.a. not applicable.

Supplementary Table S5 AICE and DAICE description. Upper case letters represent strictly identical sequences between the direct repeats. Genome accession numbers of the eleven strains of the population (chromosome and plasmids) are between CP041650-CP041654 and CP041601-CP041613 (Tidjani et al., 2019a).

Supplementary Table S6 Amino acid identity matrices of mobility signature proteins in AICE families. The identity in amino acids between the different representative of an AICE/DAICE family is indicated as a percentage. The percentages written in red indicated an identity value below 95%. Int: integrase; Rep: replicase, Abs: absence of the coding gene in the element.

Supplementary Table S7 BlastP annotations of AICE/DAICE cargo genes.

Supplementary Table S8 BlastP annotations of ICE01 cargo genes.
