# Supplementary figures and images for: Prevalence and mobility of integrative and conjugative elements within a Streptomyces natural population
Source: Front Microbiol. 2022 Sep 13;13:970179. doi: 10.3389/fmicb.2022.970179 (PMC9513070; doi:10.3389/fmicb.2022.970179)

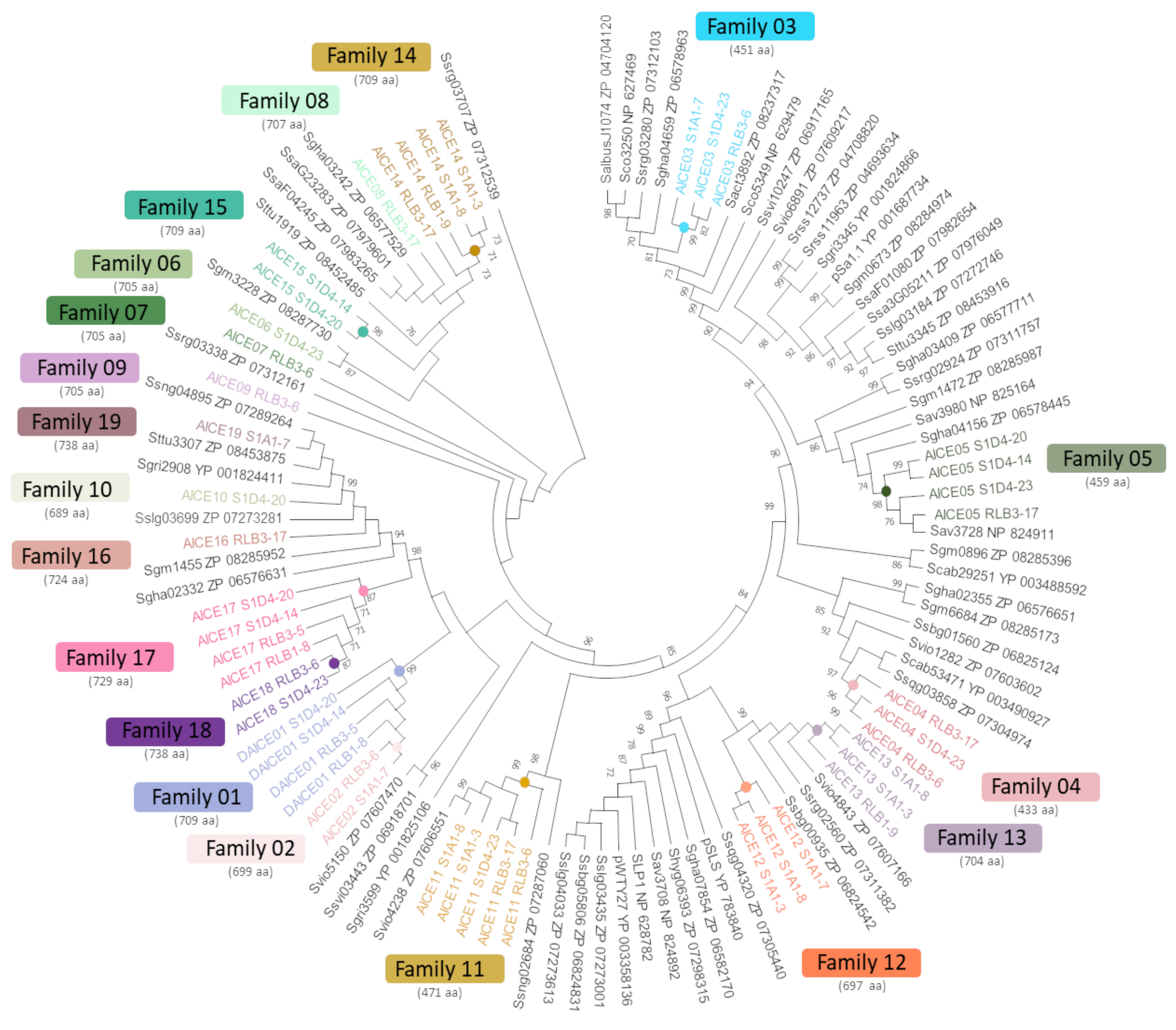

Supplement: Supplementary Figure S1 — Phylogenetic tree of TraB proteins. A tree was built with 47 out of 51 TraB proteins identified in AICEs and DAICEs of the Streptomyces population. They were compared with 62 TraB sequences retrieved from Ghinet et al. (2011) that represent the TraB diversity found in sequenced Streptomyces species. TraB sequences of the population in the tree ranged from 433 to 738 amino acids. The four excluded sequences in the tree corresponded to TraB protein of family 18, which was too divergent and four very short sequences (139 amino acids) that could not be readily aligned. Plasmid-borne TraB (n = 4) were also excluded from the analysis. The unrooted phylogenetic tree was built with full length proteins using a Neighbor Joining method (JTT model) and percentage bootstrap support (100 replicates) is indicated if >70%. All positions with <50% site coverage were eliminated leaving a total of 567 positions in the final dataset. TraB sequences from our study are indicated with colored fonts. Their affiliation to different families, based on their phylogeny and sequence identity is indicated on the tree. The reference sequences are labeled with their accession numbers and according to the nomenclature of (Ghinet et al., 2011). [file Data_Sheet_1.ZIP › Supp Mat Choufa et al. 270722/FigureS1.tif]

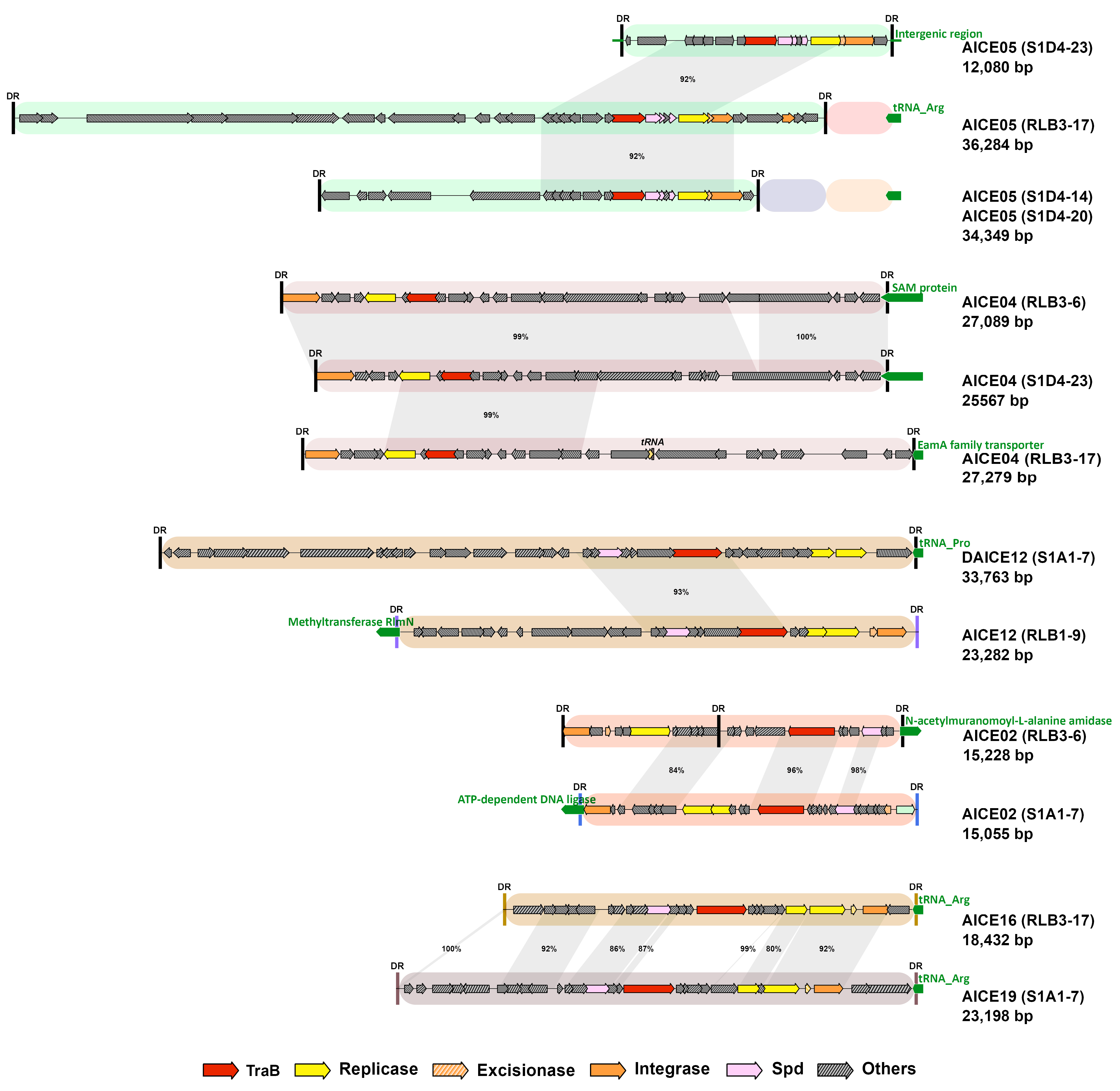

Supplement: Supplementary Figure S1 — Phylogenetic tree of TraB proteins. A tree was built with 47 out of 51 TraB proteins identified in AICEs and DAICEs of the Streptomyces population. They were compared with 62 TraB sequences retrieved from Ghinet et al. (2011) that represent the TraB diversity found in sequenced Streptomyces species. TraB sequences of the population in the tree ranged from 433 to 738 amino acids. The four excluded sequences in the tree corresponded to TraB protein of family 18, which was too divergent and four very short sequences (139 amino acids) that could not be readily aligned. Plasmid-borne TraB (n = 4) were also excluded from the analysis. The unrooted phylogenetic tree was built with full length proteins using a Neighbor Joining method (JTT model) and percentage bootstrap support (100 replicates) is indicated if >70%. All positions with <50% site coverage were eliminated leaving a total of 567 positions in the final dataset. TraB sequences from our study are indicated with colored fonts. Their affiliation to different families, based on their phylogeny and sequence identity is indicated on the tree. The reference sequences are labeled with their accession numbers and according to the nomenclature of (Ghinet et al., 2011). [file Data_Sheet_1.ZIP › Supp Mat Choufa et al. 270722/FigureS2.tif]

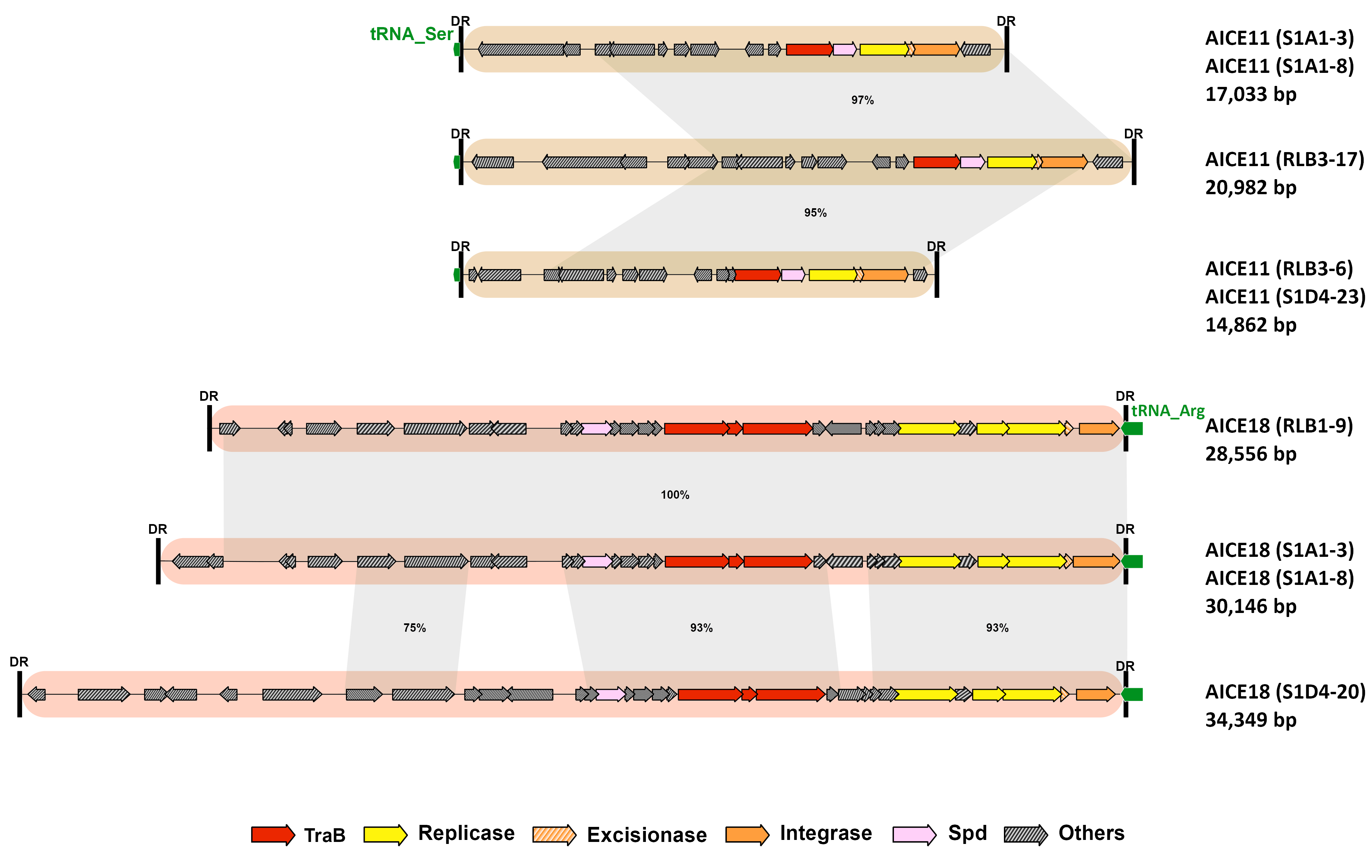

Supplement: Supplementary Figure S1 — Phylogenetic tree of TraB proteins. A tree was built with 47 out of 51 TraB proteins identified in AICEs and DAICEs of the Streptomyces population. They were compared with 62 TraB sequences retrieved from Ghinet et al. (2011) that represent the TraB diversity found in sequenced Streptomyces species. TraB sequences of the population in the tree ranged from 433 to 738 amino acids. The four excluded sequences in the tree corresponded to TraB protein of family 18, which was too divergent and four very short sequences (139 amino acids) that could not be readily aligned. Plasmid-borne TraB (n = 4) were also excluded from the analysis. The unrooted phylogenetic tree was built with full length proteins using a Neighbor Joining method (JTT model) and percentage bootstrap support (100 replicates) is indicated if >70%. All positions with <50% site coverage were eliminated leaving a total of 567 positions in the final dataset. TraB sequences from our study are indicated with colored fonts. Their affiliation to different families, based on their phylogeny and sequence identity is indicated on the tree. The reference sequences are labeled with their accession numbers and according to the nomenclature of (Ghinet et al., 2011). [file Data_Sheet_1.ZIP › Supp Mat Choufa et al. 270722/FigureS3.tif]

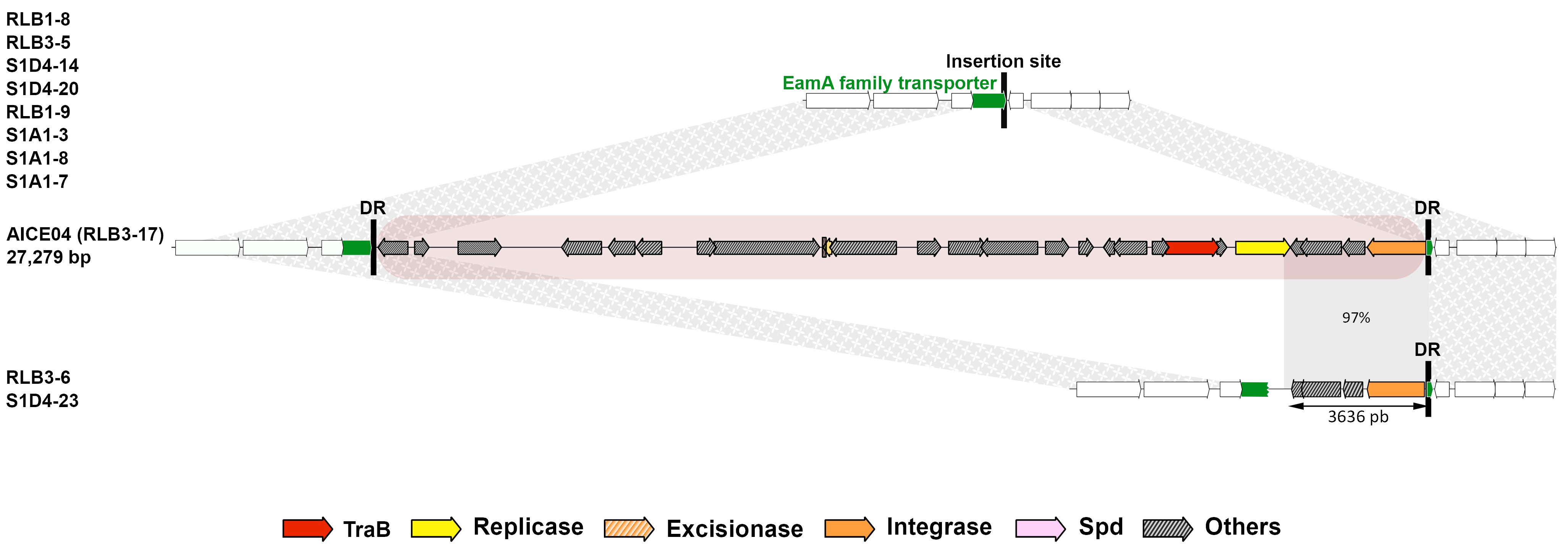

Supplement: Supplementary Figure S1 — Phylogenetic tree of TraB proteins. A tree was built with 47 out of 51 TraB proteins identified in AICEs and DAICEs of the Streptomyces population. They were compared with 62 TraB sequences retrieved from Ghinet et al. (2011) that represent the TraB diversity found in sequenced Streptomyces species. TraB sequences of the population in the tree ranged from 433 to 738 amino acids. The four excluded sequences in the tree corresponded to TraB protein of family 18, which was too divergent and four very short sequences (139 amino acids) that could not be readily aligned. Plasmid-borne TraB (n = 4) were also excluded from the analysis. The unrooted phylogenetic tree was built with full length proteins using a Neighbor Joining method (JTT model) and percentage bootstrap support (100 replicates) is indicated if >70%. All positions with <50% site coverage were eliminated leaving a total of 567 positions in the final dataset. TraB sequences from our study are indicated with colored fonts. Their affiliation to different families, based on their phylogeny and sequence identity is indicated on the tree. The reference sequences are labeled with their accession numbers and according to the nomenclature of (Ghinet et al., 2011). [file Data_Sheet_1.ZIP › Supp Mat Choufa et al. 270722/FigureS4.tif]

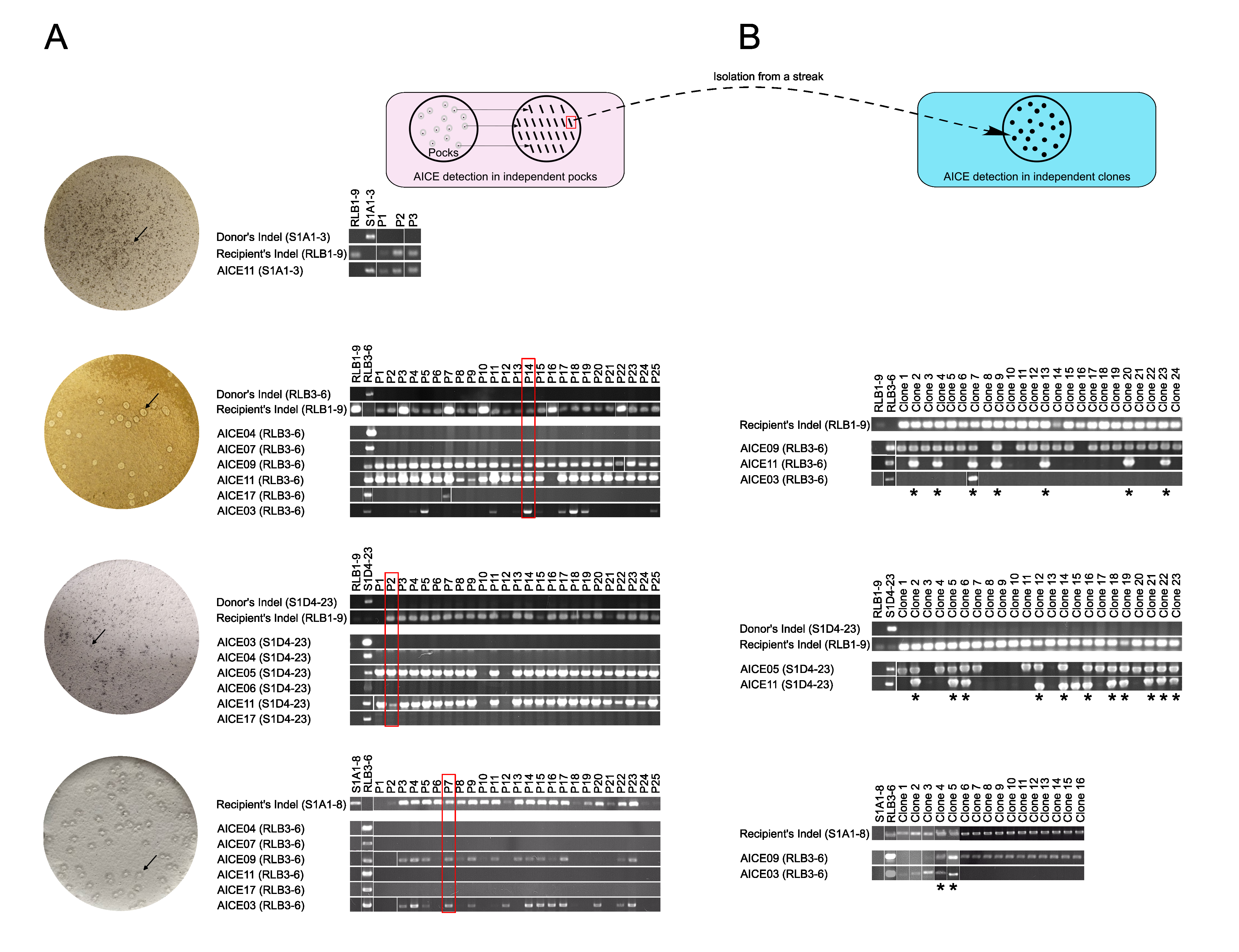

Supplement: Supplementary Figure S1 — Phylogenetic tree of TraB proteins. A tree was built with 47 out of 51 TraB proteins identified in AICEs and DAICEs of the Streptomyces population. They were compared with 62 TraB sequences retrieved from Ghinet et al. (2011) that represent the TraB diversity found in sequenced Streptomyces species. TraB sequences of the population in the tree ranged from 433 to 738 amino acids. The four excluded sequences in the tree corresponded to TraB protein of family 18, which was too divergent and four very short sequences (139 amino acids) that could not be readily aligned. Plasmid-borne TraB (n = 4) were also excluded from the analysis. The unrooted phylogenetic tree was built with full length proteins using a Neighbor Joining method (JTT model) and percentage bootstrap support (100 replicates) is indicated if >70%. All positions with <50% site coverage were eliminated leaving a total of 567 positions in the final dataset. TraB sequences from our study are indicated with colored fonts. Their affiliation to different families, based on their phylogeny and sequence identity is indicated on the tree. The reference sequences are labeled with their accession numbers and according to the nomenclature of (Ghinet et al., 2011). [file Data_Sheet_1.ZIP › Supp Mat Choufa et al. 270722/FigureS5.tiff]
